# Antimicrobial, Antiviral and Immunomodulatory Activity Studies of *Pelargonium sidoides* (EPs^®^ 7630) in the Context of Health Promotion

**DOI:** 10.3390/ph4101295

**Published:** 2011-10-10

**Authors:** Herbert Kolodziej

**Affiliations:** Institute of Pharmacy, Pharmaceutical Biology, Freie Universität Berlin, Koenigin-Luise-Str. 2+4, Berlin 14195, Germany; E-Mail: kolpharm@zedat.fu-berlin.de; Tel.: +49-30-838-53731; Fax: +49-30-838-53729

**Keywords:** *Pelargonium sidoides*, antibacterial, antiviral, immunomodulation

## Abstract

*Pelargonium* species contribute significantly to the health care of a large population in the Southern African region, as part of a long-standing medical system intimately linked to traditional healing practices. Most notably, extracts of the roots of *P. sidoides* have commonly been applied for the treatment of dysentery and diarrhoea but only occasionally for respiratory complaints. Clinical trials have shown that a modern aqueous-ethanolic formulation of *P. sidoides* extracts (EPs^®^ 7630) is an efficacious treatment for disorders of the respiratory tract, for example bronchitis and sinusitis. It should be noted that EPs^®^ 7630 is the most widely investigated extract and therefore is the focus of this review. In order to provide a rationale for its therapeutic activity extracts have been evaluated for antibacterial activity and for their effects on non-specific immune functions. Only moderate direct antibacterial capabilities against a spectrum of bacteria, including *Mycobacteria* strains, have been noted. In contrast, a large body of *in vitro* studies has provided convincing evidence for an anti-infective principle associated with activation of the non-specific immune system. Interestingly, significant inhibition of interaction between bacteria and host cells, a key to the pathogenesis of respiratory tract infections, has emerged from recent studies. In addition, antiviral effects have been demonstrated, including inhibition of the replication of respiratory viruses and the enzymes haemagglutinin and neuraminidase. Besides, an increase of cilliary beat frequency of respiratory cells may contribute to the beneficial effects of *P. sidoides* extracts. This example provides a compelling argument for continuing the exploration of Nature and traditional medical systems as a source of therapeutically useful herbal medicines.

## 1. Introduction

**Figure 1:**
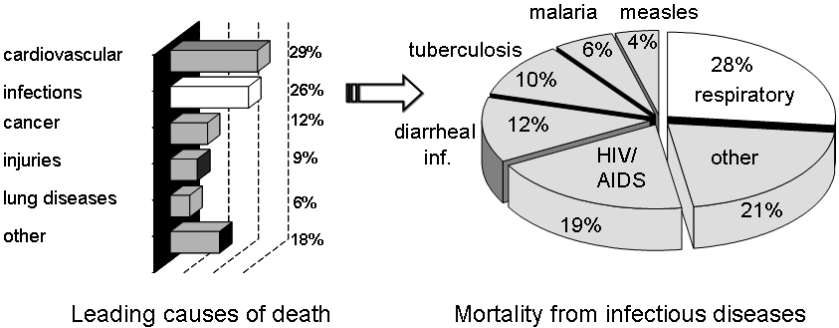
Global deaths and infectious diseases.

Infectious diseases have been and continue to be an ever-present threat to mankind. Despite medical advances, infectious diseases are still a cause of death for millions around the World. Of the estimated 57 million deaths that occur throughout the World each year, more than 25 percent are directly caused by infectious diseases [1]. More than 90 percent of the worldwide mortality from infectious diseases is caused by only a handful of diseases, including lower respiratory infections, HIV/AIDS, diarrheal diseases, tuberculosis, malaria and measles (Figure 1) [1]. New targets for novel anti-infective drugs need to be identified, particularly in the light of the emergence of drug resistant strains.

Many herbal medicines have ethnomedicinal uses for the treatment of infectious conditions such as respiratory ailments. However, research programmes are important for validating the traditional use, providing evidence for the safety and the quality of the plant material. That traditionally used herbal medicines may well provide the basis for the development of a modern and highly successful phytopharmaceutical that meets the internationally required criteria of quality, safety and efficacy for an evidence-based therapy is exemplarily shown for *Pelargonium sidoides*. Members of the genus *Pelargonium* are highly valued by traditional healers and the South African native population for their curative and palliative effects in the treatment of gastrointestinal disorders, while interest in *P. sidoides* has been heightened by reports of therapeutic benefits in infectious conditions of the respiratory tract such as tuberculosis and related diseases. The interesting ethnobotanical and commercial history, as well as the phytochemical profile of this medicinal plant, have been reviewed in detail elsewhere and are thus not reiterated (*vide infra*). This report is aimed at reviewing the current knowledge on the mechanisms of the underlying beneficial effects of *P. sidoides* and the related herbal drug preparation EPs^®^ 7630. Notably, this special extract has been the subject of 20 clinical studies involving more than 9,000 patients, including 3,900 children as young as 1 year, reviewed repeatedly elsewhere (*vide infra*). The objective of this review is to show the potential of a traditional plant, *P. sidoides*, for the defence against pathogens and the improvement of immune functions at various levels and, thus, the invaluable role that natural products continue to play in the drug discovery process in the areas of infectious and other diseases.

## 2. Discussion

### 2.1. Traditional and Therapeutic Uses of Pelargonium Sidoides

Historically, the ethnomedicinal use of *P. sidoides* in the traditional medical systems of southern Africa is mainly associated with gastrointestinal disorders (diarrhoea and dysentery), whereas only some records exist for the treatment of respiratory conditions (tuberculosis, coughs, colds, sore throat) and sources of this information are not always provided [2-5]. In this context it appears important to note the general problems surrounding the taxonomic classification of *Pelargonium* species, as reflected in the past by numerous revisions. In southern Africa, tuberculosis is one of the most commonly notified diseases. In the absence of an effective chemotherapy in earlier days, traditional healers were widely consulted and many local plants were used to alleviate tuberculosis-related symptoms. As for *P. sidoides*, a key role in the successful introduction of a profound traditional herbal remedy in modern phytotherapy in Europe is linked to Charles Henry Stevens, who believed he was cured by taking a brew prepared from the roots and subsequently tried to introduce “Stevens' Consumption Cure” on the market in Great Britain [5-8].

This herbal treatment is seeing increasing popularity, not only in southern Africa but also in European countries, in the Commonwealth of Independent States, the Baltic States and in Mexico [4,9]. Following the well-documented therapeutic benefits in infectious conditions of the respiratory tract, a modern formulation of the roots of *P. sidoides*, EPs^®^ 7630 (exclusively contained in Umckaloabo^®^, marketed by Spitzner Arzneimittel, Ettlingen, Germany), has been elaborated from this traditional herbal medicine. EPs^®^ 7630 is a special aqueous ethanolic extract. Results from a number of clinical studies including several randomised, double-blind and placebo-controlled trials, support the use of Umckaloabo^®^ in respiratory tract infections such as bronchitis, sinusitis and tonsillitis. Several post-marketing surveillance studies are available, demonstrating the safety and tolerability of the traded product [10-18]. Umckaloabo^®^ was approved in Germany as a drug for treating acute bronchitis. This illness is one of the most frequent diagnoses in medical practice and predominantly caused by RNA viruses. Only in less than 10% of cases non-viral agents play a role in its etiology [19]. Surprisingly, prescription of antibiotics for infections of viral origin is still wide-spread even though their efficacy is limited to bacterial infections [20]. One purpose might be to avoid a secondary bacterial superinfection with group A β-hemolytic streptococci. Development of resistant bacteria is a disadvantage of non-indicated antibiotic therapy. Based on clinical studies, the *Pelargonium*-based preparation offers an alternative therapeutic agent to otherwise not indicated antibiotic treatment.

### 2.2. Antimycobacterial and Respiratory Tract Related Antibacterial Activity

Following earlier claims of antimycobacterial efficacy of *P. sidoides*, methanol extracts of the roots and some of its characteristic phenol and coumarin constituents were tested for this particular biological activity. Using the radiorespirometric BACTEC method, the extracts showed antimycobacterial activity against *Mycobacterium tuberculosis* (96% at 12.5 μg/mL), while none of the purified compounds tested exhibited any antimycobacterial activities under these experimental conditions. The same extract exhibited weak activities against *M. tuberculosis* (MIC of 100 μg/mL) as assessed in a broth microdilution alamar blue assay. Rifampicin, used as reference, showed an MIC of 0.06 μg/mL under the experimental conditions used. Independent support of the weak antimycobacterial activity of *P. sidoides* was provided by similar studies of acetone and ethanol root extracts using *M. tuberculosis* and *M. smegmatis* as test organisms, while coumarins and flavonoids isolated from this plant source again proved inactive [4]. With MICs of >1,024 μg/mL, an aqueous acetone extract of *P. sidoides* showed negligible antimycobacterial activities against the rapidly growing avirulent *M. smegmatis* in a broth microdilution assay [21]. Similar results were obtained from testing the extracts against *M. aurum*, a species reported to be predictive of activity against *M. tuberculosis* because of their comparable drug sensitivity profiles. This finding suggests that direct antimycobacterial activity apparently depends not only on the strain of mycobacterium, but also strongly on extract preparation and composition.

In the course of a search for antimycobacterial constituents some straight-chain fatty acids present in *n*-hexane root extracts with activity against a panel of rapidly growing mycobacteria were identified [22,23]. Interestingly, all the saturated fatty acids except lauric acid were devoid of any antimycobacterial activity, whereas unsaturated analogues, including oleic acid and linoleic acid, were active, with the latter being the most potent one (MIC of 2 μg/mL against *M. aurum*). However, these data do not provide convincing evidence for an antimycobacterial principle of *P. sidoides*. In traditional medicine decoctions and infusions are used, thus putting into question the presence of effective levels of lipophilic constituents in aqueous preparations. Furthermore, it should be noted that *Mycobacteria* are intracellularly residing pathogens, while the *in vitro* protocols conducted reflect a direct action on these pathogens. That *P. sidoides* root extracts apparently show limited antimycobacterial activity under these test conditions is reflected in further studies [24,25]. Taking into account the overall moderate antimycobacterial activity in experiments carried out so far, it is highly likely that the claimed effective use of *P. sidoides* in the treatment of tuberculosis may be due to stimulation of the non-specific immune system rather than any direct action on *Mycobacteria* pathogens. The antimycobacterial principle of this popular *Pelargonium* species remains to be identified.

Parallel work focused on the evaluation of the antibacterial activity of P. sidoides against a panel of microorganisms (*Staphylococcus aureus, Streptococcus pneumoniae, ß-hemolytic Streptococcus, Escherichia coli, Klebsiella pneumoniae, Proteus mirabilis, Pseudomonas aeruginosa, Haemophilus influenzae*) associated with respiratory diseases [26]. However, the *in vitro* antibacterial effects of an aqueous acetone extract, fractions obtained thereof from successive partitions between water and organic solvents (ethyl acetate, n-butanol, MICs of 600–7,500 μg/mL), and a range of isolated compounds (MICs of 200–1,000 μg/mL) have been shown to be only modest compared with classic antibiotics. It should be noted that the standardized root extract EPs^®^ 7630 has hitherto not been subjected to similar antibacterial studies, though moderate activities are commonly noted. Independent support of weak direct antibacterial activities followed from studies using the liquid preparation Umca^®^ and isolates (*Streptococcus pneumonia, S. pyogenes, S. viridans, Staphylococcus aureus, S. epidermis, Neisseria* spp., *Moraxella catarrhalis, Haemophilus influenza*) obtained from the throat cultures of patients [27]. Employing a microdilution broth method, MIC values were in the range of 200–1,600 μg/mL, with MICs >800 μg/mL for most of the bacteria tested.

These disappointing findings prompted a series of studies attempting to demonstrate indirect antibacterial mechanisms that could provide a clue to the anti-infective properties of *P. sidoides*-based herbal medicines [28]. Adhesion of pathogenic bacteria to the host cell surface is a crucial event in colonization and infection. Inhibition of the microbial docking process to epithelial cells at an early stage would be effective in protecting host cells from infection. Using fluorescent-labelled group A-streptococci and viable human laryngeal cells (HEp-2) as a model, flow cytometric measurements showed prominent (by up to *ca.* 45% compared with untreated cells) anti-adhesive and anti-invasive capabilities of EPs^®^ 7630 in a concentration dependent manner (< 30 μg/mL). Notably, inhibition of streptococcal adhesion to host cells was only evident when the bacteria were pre-treated with EPs^®^ 7630, indicating that the interaction of the anti-adhesive principle occurred only with the bacterial outer membane surface and not with binding sides at the epithelial surface.

**Figure 2:**
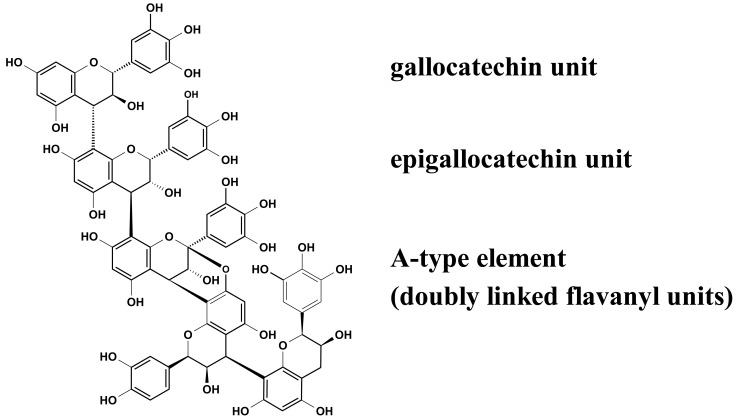
Representative structure of the proanthocyanidins of *Pelargonium sidoides* with epigallocatechin and gallocatechin extender units as well as doubly linked A-type elements.

Recent experiments revealed that proanthocyanidins (Figure 2) play a decisive role in the observed inhibition of group A-streptococci docking process to HEp-2 cells, as concluded from the apparent ‘missing’ activity of phenolic-free extracts [29]. The picture that emerged from these anti-adhesive studies including homogeneous epicatechin- and catechin-based polyflavans, a ‘mixed’ procyanidin sample, a prodelphinidin test substance as well as an A-type proanthocyanidin provided evidence that the anti-streptococcal activity of EPs^®^ 7630 polyphenols appeared to be due to the prodelphinidin-type substructure (epigallocatechin and gallocatechin extender units). Pre-treatment experiments indicated that the interaction of the anti-adhesive proanthocyanidins occurred only with binding factors on the bacterial surface. The blocked molecules, for example fibronectin- and collagen-binding adhesions [30], remain to be identified.

Interestingly, bacterial attachment was enhanced *ca.* 7-fold under the influence of EPs^®^ when using buccal epithelial cells that, noticeably, are mostly dead [28]. Inactivation of the trapped microorganisms may be explained by the fact that the decaying buccal cells are physiologically sloughed off and swallowed with the saliva. In this way the intercepted pathogens are not available for adhesion to intact cells. Taking into account the fairly high inhibition of bacterial adhesion to viable epithelial cells, these indirect antibacterial activities have beneficial effects on infectious conditions. The impact of EPs^®^ 7630 on microbial adhesion was also demonstrated in studies with *Helicobacter pylori* [31,32]. Again, pre-treatment of fluorescent-labelled *H. pylori* with the *P. sidoides* extract inhibited the adherence to human gastric mucosa cells in a dose-dependent manner. However, the underlying biologically active principle remains to be identified. Taken together, the demonstrated anti-adhesive mechanism may well contribute to the anti-infective activity of EPs^®^ 7630 at an early time point of a bacterial infection.

Also worthy of mention is the potential of the *P. sidoides* extract to significantly inhibit streptococcal invasion into HEp-2 cells. Similarly to the anti-adhesion activity, polyphenols may interfere with extracellular proteins produced by the bacteria to facilitate invasiveness. Clearly, further studies are needed to get insight into the underlying principle that bacteria employ to invade our cells. From a more medicinal viewpoint, inhibition of the bacterial invasion of epithelial cells protects the host from pathogens that evade host defences and antibiotic therapy. Once inside the host cells, recurrent streptococcal infections may well occur. The demonstrated inhibition of the streptococcal invasion thus prevents recurrent infections.

Although the above findings have provided evidence for both direct and indirect antibacterial properties of *P. sidoides*, the pronounced anti-infective capabilities of this herbal medicine cannot adequately be explained merely on the basis of the observed antibacterial activities.

### 2.3. Effect on the Mucociliary System of Respiratory Cells

The mucociliary system represents a defence mechanism of the nasal cavity and the bronchial tree for cleaning the air of bacteria and foreign particles, with the ciliary beat frequency (CBF) being an important parameter for determining its efficacy. EPs^®^ 7630 significantly and concentration-dependently increased CBF of an adherent monolayer culture of human nasal epithelium cells. At 30 μg/mL, the increase was *ca.* 125% compared to the equilibrium phase [33]. After washing procedures the CBF returned to that of the equilibration period. However, subsequent examinations have to prove the effect under *in vivo* conditions with emphasis on the mucociliary clearance.

### 2.4. Antiviral Effects

Since respiratory tract infections are frequently caused by viruses (> 90% of cases) [19], it is noteworthy that EPs^®^ 7630 exhibited antiviral effects in a number of assays. For example, prominent cytoprotective effects were observed in a fibroblast-virus protection assay [34]. In this test model, supernatants of activated macrophages were transferred to a monolayer of encephalomyocarditis virus (EMCV)- and IFN-sensitive murine L 929 fibroblasts (ML929F) before exposure to EMCV suspensions. IFN activity was assessed by observing the protection of ML929F against the cytopathic effect (CPE) induced by infection with EMCV. The relative number of viable cells was determined spectrophotometrically using crystal violet as staining reagent for protected cells [35]. Antiviral protection was expressed in U/mL, defined as the reciprocal value of the supernatant dilution that would inhibit 50% of the CPE induced by EMCV in ML929F. These values were correlated with the IFN standard (100 U/mL) to account for any fluctuation in assay sensitivity. Prominent cytoprotective effects (*ca.* 60%) were observed for EPs^®^ 7630 at remarkably low doses (0.8 μg/mL), with complete inhibition of CPE at 1.4 μg/mL sample concentration [34]. Recent refined work showed that the supernatant of bone marrow-derived macrophages (BMMΦ) stimulated with 10 μg/mL of EPs^®^ 7630 produced an antiviral activity of *ca.* 80 U/mL, which was similar to that of the LPS (1 ng/mL) response [36]. Known antiviral factors are members of the IFN family classified in type I and type II interferons according to receptor specificity and sequence homology [37,38]. Since the functional assay does not discriminate between IFN-α, IFN-β and IFN-γ, the authors' interest focused on their detection using ELISA. All attempts to identify any potential antiviral compound were unsuccessful, suggesting that either the production of effective IFN levels was below the detection limits of the assays, synergistic effects may contribute to the powerful overall action or other elements of the immune system such as type III IFN [39] are the responsible effective agents. That eicosanoids may play a role in the control of viral infection may be excluded. EPs^®^ 7630 was shown to suppress their production in calcium-ionophore stimulated human granulocytes [40]. Previous studies with a range of proanthocyanidins indicated that members of this class of compounds possess pronounced IFN-like activities [41]. Their presence in the extract may also be an inducing factor. Worthy of mention is that the extract did not induce any cytoprotective effects when directly exposed to ML929F, clearly indicating an indirect mode of action.

As noted, known antiviral factors are members of the IFN family. The apparent modulatory potency of EPs^®^ 7630 on IFN levels, endowed with potent antiviral effects and mediating functional activity of natural killer cells [42], was demonstrated by enhanced IFN-ß synthesis in human MG-63 osteosarcoma cells when superinduced with the viral double-stranded RNA analogue polyinosinic acid: polycytidylic acid [35]. The effect was only evident in the presence of the infectious component, with the maximum augmentation of 200% after application of 3 μg/mL. This remarkable selectivity is significant in that activation of the non-specific immune system comes into force only after invasion by pathogens.

Direct antiviral activity of the *P. sidoides* aqueous extract was first evaluated *in vitro* against herpes simplex virus type 1 (HSV-1) and type 2 (HSV-2) on RC-37 cells using a plaque reduction assay [43]. Both herpes viruses cause common infections with recurrent orofacial (HSV-1) and genital lesions (HSV-2). Only pretreatment of viruses with the extract and subsequent infection of RC-37 cells revealed pronounced antiviral effects in a concentration-dependent manner, with IC_50_ values of 0.6 μg/mL and 0.05 μg/mL for HSV-1 and HSV-2, respectively. Taking into account the excellent selectivity index defined as the ratio of IC_50_ values between the cytotoxicity on RC-37 cells (0.04 mg/mL) and the antiherpes activity, the antiviral effect is likely due to interactions between the virus and extract components which, however, remain to be identified. Notably, at the maximal noncytotoxic sample concentration of 10 μg/mL, plaque formation was reduced by *ca.* 99.9% for HSV-1 and HSV-2. The mode of action was investigated by a set of experiments with incubation during the adsorption or replication period of the virus. The results showed that addition of the extract exclusively during the adsorption period of HSV significantly reduced infectivity. Again, polyphenols may represent the underlying active principle, acting in a selective rather than non-specific manner.

Following the therapeutic use of EPs^®^ 7630 in modern phytomedicine for the treatment of upper respiratory tract infections, the demonstrated antiviral effects of this extract against a panel of viruses including seasonal influenza A virus strains (H1N1, H3N2), respiratory syncytial virus, human coronavirus, parainfluenza virus and coxsackie virus provided evidence of feasible beneficial effects in patients suffering from acute bronchitis and related infectious diseases [44]. With the exception of coxsackie, all sensitive viruses are enveloped, suggesting that EPs^®^ 7630 inhibits preferably the replication of viruses of this category. This was in line with the potential antiviral effects on enveloped herpes viruses [43]. In fact, EPs^®^ 7630 did not show any influence on non-enveloped rhino- and adenoviruses-induced cytopathogenic effects. Observed varying susceptibilities to EPs^®^ 7630 of influenza A subtypes (H1N1, H3N1, H5N1) can be rationalized by mutant haemagglutinins present on the surface of the individual influenza viruses. These structurally modified glycoproteins, responsible for the binding of the virus to the host cell, may therefore be differently affected by the extract. Experiments evaluating the influence of EPs^®^ 7630 on virus-induced cytopathogenic effects in a range of cell lines indicated IC_50_ values > 100 μg/mL.

The enzyme neuraminidase (sialidase) with its highly conserved active site plays a key role not only in the release of virions from infected host cells, but also in their movement through the upper respiratory tract [45]. Among the therapeutic options for the treatment and prevention of influenza infections, neuraminidase inhibitors are promising candidates to inhibit the spread of the viral infections. In view of the claimed antiviral potential related to common colds, EPs^®^ 7630 was originally tested for its *in vitro* neuraminidase inhibiting activity using a fluorometric-based assay [46]. Compared to zanamivir (IC_50_ of 71 μg/mL), the extract exhibited pronounced inhibitory activity for the bacterial neuraminidase from *Vibrio cholera*, with an IC_50_ value of 0.9 μg/mL [47]. The evidence, however, that EPs^®^ 7630 has similar inhibiting activity for the viral neuraminidase in this *in vitro* model demands further investigation. Even more interesting, treatment of the extract with skin powder for the removal of tannins produced an inactive sample. The loss of activity of the polyphenol-free extract clearly indicated that proanthocyanidins apparently represent the underlying active principle. The specificity of EPs^®^ 7630 polyphenols for this biological activity was demonstrated by testing a range of proanthocyanidin samples comprised of different flavanyl constituent units (procyanidins, prodelphinidins, A-type proanthocyanidins).The data suggested that the neuraminidase inhibiting activity of EPs^®^ 7630 may be related to prodelphinidin extender units in oligoflavanoids.

Independent support of the antiviral activity of EPs^®^ 7630 originated from a current study using influenza A strains H1N1, an oseltamivir-resistant H1N1 subtype and H3N2 [48]. The data suggest inhibition of the key proteins, haemagglutinin and neuraminidase, present on the surface of the influenza virus. This potency was also evident in *in vivo* experiments with the H1N1 virus. Considering the interaction of EPs^®^ 7630 constituents with the two surface glycoproteins that are important for the viral replication lifecycle at the early and final stage of infection, the therapeutic benefit of the extract may well be seen in the light of an infect prophylaxis and improvement in the infectious condition.

### 2.5. Role of the Non-Specific Immune System

**Figure 3:**
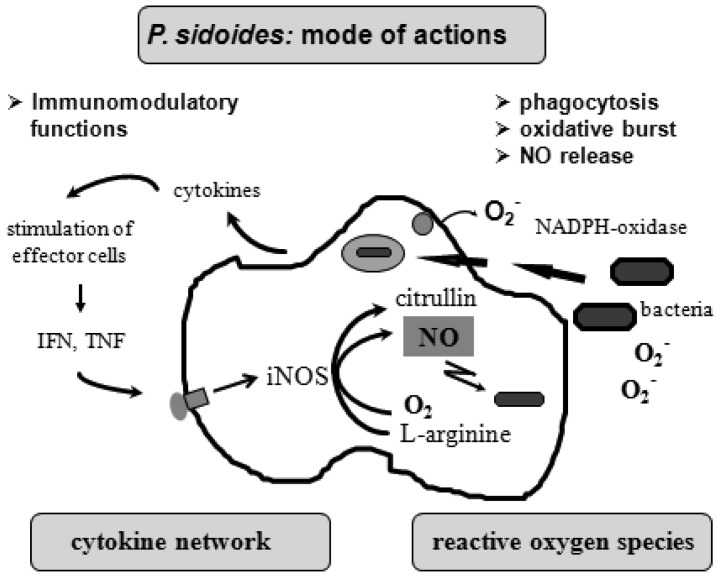
Simplified illustration of cytotoxic defence mechanisms of activated macrophages induced by the root extract of *P. sidoides*.

To understand the many effects of *P. sidoides* on the non-specific immune system and its potential role in host response to pathogens, it seems appropriate to illustrate the key components involved in the clearing of microbial agents. Since several immunological factors are associated with anti-infective responses, only those parameters are briefly described which primarily imply stimulation of the non-specific immune system and are closely related to the hitherto known anti-infective principle detected in *P. sidoides* based medicines. A simplified overview of induced cytotoxic defence mechanisms is shown in Figure 3.

Macrophages are extremely versatile cells involved in a number of complex processes in immune responses. When activated they acquire microbicidal effector functions and secrete cytokines, resulting in recruitment of immune cells and subsequent elimination of the pathogen by phagocytosis or release of reactive oxygen and nitrogen species. Generation of reactive oxygen species is initiated by NADPH oxidase. The primary product is superoxide, which can be converted to H_2_O_2_ by superoxide dismutase and subsequently to hydroxyl radicals and anions.

Production of nitric oxide and its many congeners (NO) is another key macrophage antimicrobial response. NO are important intra- and intercellular regulatory molecules of multiple biological functions, including macrophage-mediated cytotoxicity [49-52]. Endogenously derived NO is generated enzymatically from L-arginine by constitutive (c) or inducible (i) nitric oxide synthases (NOS) present in different cell types and released constantly at physiological levels under the influence of cNOS [53]. All NO synthases convert L-arginine and molecular oxygen to L-citrulline and NO. However, expression of iNOS in activated macrophages in response to immunological stimuli induces the release of large amounts of NO for long periods. Although NO species function beneficially as antimicrobial effector molecules in the immune protection against bacteria, parasites and viruses and regulate cell survival, the relatively high and sustained level of inducible NO produced during the immune response may be harmful, as dramatically shown in septic shock. Accordingly, regulation of NO production is important for human health. In the presence of superoxide anion, a series of NO effector molecules might prevail that are also involved in the host defence mechanism.

Although the production of reactive oxygen and nitrogen species considerably contributes to the killing of infectious pathogens, the secretion of cytokines from activated macrophages and related cells is also an integral component of an effective immune response to a viral or bacterial infection, with regulatory functions on the production of these cytotoxic effector species. They act at all levels of the immune response and form a complex network of molecules. Among the macrophage-associated cytokines, release of tumour necrosis factor (TNF)-α is an essential early step in a signalling cascade leading to production of antimicrobial NO [54,55]. Also, TNF-α synergizes with IFN-γ in the induction of iNOS and NO production by macrophages, though IFN-γ alone was shown to be capable of independently enhancing iNOS transcription and NO release [56,57]. The role that these cytokines play in NO-mediated destruction of microbial pathogens prompted in the beginning exploration of *Pelargonium*-induced immune modulatory effects on macrophage functions using functional assays such as a fibroblast-lysis assay for release of TNF and a cytopathic effect inhibition assay for IFN-like properties (*vide infra*). Importantly, control of viruses is achieved through IFN-〈 and IFN-ß which are produced by host cells, while IFN-γ mediates macrophage activation.

### 2.6. Immunomodulatory Activities

The destabilized defence mechanisms resulting from a viral infection can clear the way for a secondary bacterial infection. Recalling the ethnomedicinal use of the roots of *P. sidoides* in the treatment of tuberculosis, it is appropriate to stress that pathogenic mycobacteria have developed multiple mechanisms for entering macrophages by a receptor-mediated pathway that is not coupled to the activation of macrophage cytotoxic defence mechanisms to ensure their own survival [58]. The fact that mycobacteria reside within cells and the less effective direct antibacterial potencies (*vide supra*) suggested that stimulation of the non-specific immune system may contribute to the anti-infective activity of *P. sidoides* extracts. The immediate question thus concerned the factors that contribute to the immune control of microbial pathogens and possibly viruses associated with respiratory tract infections.

#### 2.6.1. Phagocytic Activity and Oxidative Burst

Phagocytosis and related activity of phagocytes play a crucial role in the innate immunity of host defence against invading pathogens including bacteria and viruses. Circulating blood phagocytes are rapidly recruited to sites of infection. Activated by attached pathogen-associated molecular patterns, the microorganisms are ingested by the phagocytes (phagocytosis) followed by the production and release of reactive oxygen species (oxidative burst) that contribute to the destruction of pathogens (intracellular killing). Using a whole blood-based, flow cytometric assay with *Candida albicans* as the target organism, EPs^®^ 7630 significantly increased the number of phagocytosing cells (maximal enhanced by 56% after 2 min upon addition of 30 μg/mL of the extract sample) in a concentration-dependent manner. With a target to effector cell ratio of 1:1, all yeast cells were ingested after 30 min [59].

As for the oxidative burst, the application of 30 μg/mL extract similarly led to a marked increase of burst-active blood phagocytes for all time points observed beyond 2 min, with a maximum augmentation of 120% at 4 min when compared with controls. The stimulation of the burst activity was still detectable even when all *Candida* organisms have already been ingested. Although phagocytic activity may strongly depend on the kind of microorganism, this finding clearly demonstrated that the *Pelargonium* extract effectively improved phagocytic activity. Furthermore, results from a microbiological intracellular killing assay indicated that the number of surviving yeast cells were reduced by *ca.* 30% at 120 min compared with controls. The benefit of the oral application may be seen in local effects on both the resident and invading phagocytes at the site of infection.

#### 2.6.2. Intracellular Killing of Pathogens and Release of Nitric Oxides

**Figure 4:**
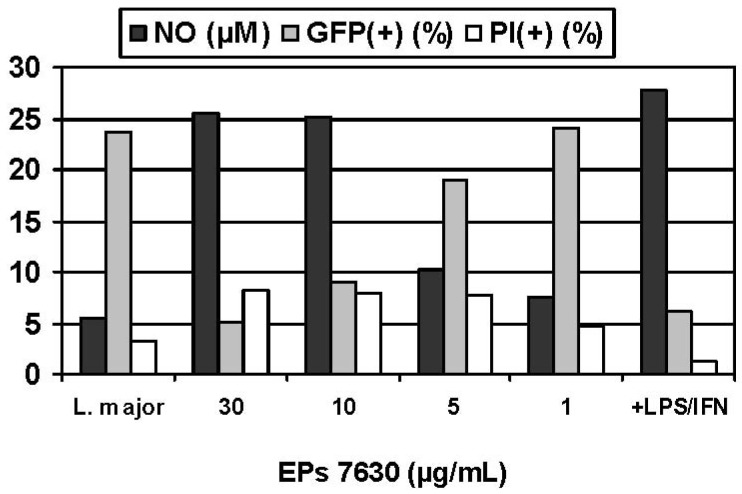
Immune response in *L. major* GFP-infected macrophages treated with EPs^®^ 7630: NO production (Griess assay), GFP-positive events (relative survival rates of parasites; FAS analysis), and propidium iodide-positive events (dead host cells; FACS analysis).

Experimental infection of macrophages constitutes a particularly versatile model for assessing the immunoregulation that occurs during a cell-mediated response to an intracellular pathogen, consistent with the previous antitubercular usage of *P. sidoides* in traditional medicine. For safety reasons and convenience, an established *in vitro* model for infectious diseases was selected in which murine macrophages were infected with obligate intracellular *Leishmania* parasites rather than pathogenic *Mycobacteria* strains [60]. A colorimetric assay was used for assessing the activation of macrophages against the intracellular parasites, based on infection of macrophages in suspension culture, exposure to the *Pelargonium* extract, and lysis of the host cells with sodium dodecyl sulfate (SDS) [61]. Surviving *Leishmania* organisms were then quantitated by their conversion of yellow 3-(4,5-dimethylthiazol-2-yl)-2,5-diphenyltetrazolium bromide (MTT) into a blue formazan product. While *Pelargonium* extracts, including parent aqueous acetone and methanol extracts as well as organic fractions (petroleum ether, ethyl acetate, *n*-butanol), proved to be inactive against extracellular promastigote forms of *Leishmania* species, pronounced antileishmanial effects were observed against intracellular amastigotes that are of clinical and pharmacological importance, with EC_50_ values ranging from < 0.1 to 3.3 μg/mL compared with that of 7.9 μg/mL for Pentostam^®^ as positive control [60]. Confirmatory evidence was recently provided by fluorescence activated cell scanning (FACS) analysis using transgenic *L. major* expressing green fluorescent protein (GFP) [62]. This method has significant advantages over those involving counting parasites in microscopic preparations and the SDS lysis/MTT protocol. Treatment of infected BMMΦ with EPs^®^ 7630 resulted in a significant decrease of the intracellular GFP signal in a dose dependent manner and reaffirmed previous results concerning the activation of immune defence mechanisms but at single cell levels (Figure 4). That reduction of the parasite load was not due to general cytotoxicity followed from addition of propidium iodide before FACS measurement in order to discriminate dead (PI-positive cells< 10% in all experiments) from living cells. Exposure of infected cells to EPs^®^ 7630 in combination with IFN-γ markedly enhanced the effects. For example, the combination of EPs^®^ 7630 (10 μg/mL) plus IFN-γ (100 U/mL) was almost as effective as IFN-γ (100 U/mL) plus LPS (10 ng/mL) in killing intracellular parasites [36].

Given the crucial role of NO as cytotoxic effector molecule (*vide supra*), the NO inducing potential of *Pelargonium* extracts and their constituents was determined using the supernatants of sample treated macrophage (BMMΦ, RAW 264.7) cultures as a source of secreted NO which was quantitated by determining the nitrite concentration using the Griess assay. That the immune response was considerably more expressed in infected cells suggests effective stimulation of the non-specific immune system when needed. Compared with the positive control (100 U/mL IFN-γ + 10 ng/mL LPS), the NO inducing potential of hydrophilic *Pelargonium* extracts was significantly less prominent. However, it should be noted that the induced NO production strongly depended on the kind of macrophages (BMMΦ; RAW 264.7) used in experimental models [63]. Inhibition of iNOS by addition of L-NMMA produced significantly lower NO levels and concomitantly increased the GFP signal, providing strong evidence for the crucial role of NO as toxic effector molecule in the host defence to microbial infections and the pronounced NO-inducing capabilities of EPs^®^ 7630. Although a strong inverse correlation between NO production and parasite kill was observed, the involvement of additional NO-independent defence mechanisms cannot be ruled out.

The above promising results prompted studies with an additional infection model using intracellular *Listeria monocytogenes* bacteria and motivated the pursuit of more relevant studies at the cellular and molecular level [64]. This experimental infection model is also commonly used for studying antimicrobial defence [65]. Consistent with the *Leishmania* model, exposure of infected cells to EPs^®^ 7630 significantly enhanced NO production in a concentration dependent manner when compared to just infected cells. Conspicuously, NO production was already increased at an early time point post infection (6 h). Although the survival rate was not measured in this FACS-based assay due to non-availability of labelled *L. monocytogenes* organisms, an efficient elimination of the invading pathogens may be anticipated. The data suggest that EPs^®^ 7630 induces NO release in a broad spectrum of infectious conditions and, importantly, in therapeutically relevant doses (≤ 30 μg/mL). Accordingly, stimulation of the non-specific immune system may be anticipated for intracellular residing mycobacteria. Although this still remains to be demonstrated, the present data provide a clue for the claimed efficacy of this herbal medicine in the earlier treatment of tuberculosis and related diseases in traditional medicine. Interestingly, when tested against macrophages as a mammalian host cell control, all samples revealed no or only moderate cytotoxicity.

#### 2.6.3. Induction of the Release of Tumour Necrosis Factor, IL-1 and IL-12

Macrophage activation usually is a polyphenotypic event, with TNF release during the early response to infection [54]. Measurement of TNF-activity induced by the above mentioned *Pelargonium* extracts was therefore another important immunological parameter in the evaluation of the anti-infective potential of this herbal medicine. Initially, TNF-release was assessed in a functional assay [66], in which TNF-sensitive ML929F treated with supernatants of sample-activated BMMΦ were rapidly lysed in its presence. Surviving cells incorporate crystal violet dye and their relative number was spectrophotometrically determined. At the concentration of ≤ 25 μg/mL, the ethyl acetate (*ca.* 20 U/mL) and the *n*-butanol phase (*ca.* 19 U/mL) were found to possess a moderate TNF-inducing potential. All remaining samples showed only a negligible effect (< 5 U/mL) compared to the LPS stimulus (10 ng/mL; 184 U/mL) as positive control [60]. Having in mind that systemic TNF can be fatal the moderate TNF-inducing capability of the *Pelargonium* extracts in non-infected cells may be beneficial. This functional bioassay has hitherto not been extended to infected macrophages. However, gene expression analysis clearly showed augmented and prolonged up-regulation of TNF- α transcripts in infected cells (*vide supra*). Confirmatory evidence of increased intracellular TNF- α levels induced by EPs^®^ 7630 in infectious conditions compared to non-infected cells was recently obtained from FACS analysis at single cell levels [64]. Conspicuously, the accumulation of this membrane-bound cytokine in stimulated infected cells (*ca.* 35%) was less pronounced than that in untreated cultures (*ca.* 45%). This phenomenon may be explained by an enhanced activity of the TNF-α converting enzyme mediated by the herbal extract. This conjecture found support by increased soluble TNF-α protein levels detected by ELISA in the supernatant of infected cells treated with EPs^®^ 7630. The TNF-α titers (*ca.* 10–16 ng/mL; EPs^®^ 7630 test concentrations 1–30 μg/mL) were conspicuously above those of both the positive LPS control and infected cells (*ca.* 10 ng/mL in each case).

Besides TNF-α, early macrophage-associated cytokine production includes interleukin (IL)-1 and IL-12. Analyses of the intracellular/membrane-bound cytokines were again carried out in parallel in non-infected and in infected cells in the absence and presence of EPs^®^ 7630, respectively, at single cell levels using flow cytometry. ELISA was used for monitoring secreted cytokines in cell supernatants. Using BMMΦ experimentally infected with *L. monocytogenes*, incubation with EPs^®^ 7630 increased the production of intra- and extracellular IL-1 and IL-12 when compared to non-infected and just infected cells [64].

The demonstrated augmented productions of IL-1, IL-12 and TNF-α confirmed once more the potential of EPs^®^ 7630 to induce host defence mechanisms for controlling infectious agents at single cell level. It is known that IL-1 synergizes with TNF-α in the induction of IL-12 production which, in turn, induces IFN-γ release from both NK cells and T cells. IFN-γ in turn mediates protection by inducing NO production. Also, the importance of IL-12 in Th1 maturation has been demonstrated, thus providing a link between innate and adaptive immunity.

### 2.7. Gene Expression Experiments

**Figure 5:**
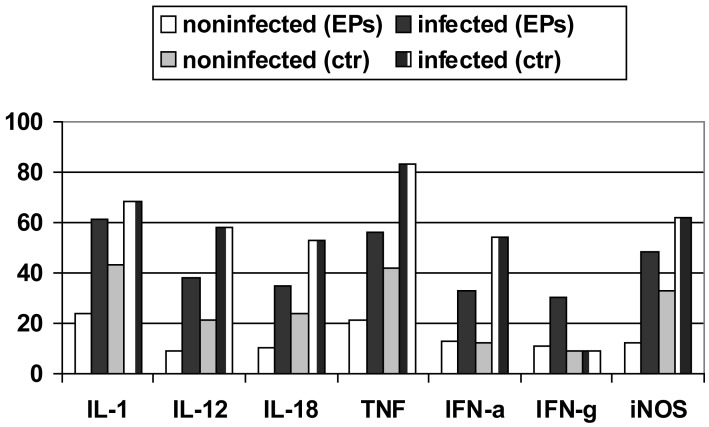
Expression of iNOS and cytokine transcripts in non-infected and in *L. major* infected RAW 264.7 cells stimulated for 4 h with 50 μg/mL of EPs^®^ 7630 and LPS/IFN-γ as positive control (ctr). Results are shown relative to hypoxanthine-guanine-phosphoribosyl transferase mRNA, defined as 100%.

Induction of protective immunity against *Leishmania* infection and other pathogenic agents is closely linked to the production of cytokines. The analysis of the data obtained from densitometric measurements revealed that the iNOS and cytokine (IL-1, IL-10, IL-12, IL-18, IFN-α, IFN-γ and TNF-α) mRNA levels of infected RAW 264.7 cells were significantly enhanced in response to LPS plus IFN-γ when compared with those of non-infected cells. Notably, EPs^®^ 7630 produced similar transcript profiles with considerably increased and prolonged up-regulations distinctly expressed under infectious conditions (Figure 5) [67-69].

Transcripts of IL-10, a cytokine associated with downregulatory functions [70], were clearly expressed later. This finding independently supported the anti-infective potential of *P. sidoides* observed at functional levels (*vide supra*). Interestingly, this herbal medicine also stimulated infected cells to produce IFN-γ transcripts. The expression of IFN-γ itself in cells of monocytic lineage has merely been noted under certain physiological and pathological conditions [71,72]. Also worthy of mention is the up-regulation of IL-12 and IL-18 mRNA levels in that both cytokines are critical to host defence against a variety of pathogens and are involved in the production of IFN-γ [73,74]. Subsequent studies suggested that IFN-γ mRNA expression appeared to be a unique feature of *P. sidoides* among herbal extracts [75]. The gene expression experiments thus provided evidence for macrophage activation at the transcriptional level.

### 2.8. Expression of Surface Markers

Recently, some surface markers became a focus of research interest [64]. CD40 is a member of the TNF receptor superfamily, expressed primarily on antigen presenting cells including macrophages. This surface marker was found to be an important regulatory receptor for IFN-γ dependent and independent host defence mechanisms [76,77] and to be essential for the development of a CD4^+^ T cell mediated immune response [78]. Stimulation of non-infected and *Listeria*-infected BMMΦ with EPs^®^ 7630 moderately enhanced the expression of CD40 [63]. However, it was suggested that this herbal medicine may sustain up-regulated CD40 expression in infected conditions as concluded from prominent expressions at the very low concentration of 1 μg/mL.

Stimulation of BMMΦ with LPS + IFN-γ or EPs^®^ 7630 (30 μg/mL) resulted in a dramatic down-regulation of the IFN-γ receptor subunit CD119 after 6 h post infection, which may be rationalized by internalisation of the receptor. The down-regulation of this receptor under infectious conditions was consistent with similar effects in *L. monocytogenes* infected BMMΦ [79]. Changes in the expression of CD119 are suggested to affect the sensitivity of the cell in responding to IFN-γ [80]. These limited molecular studies do not allow definite statements, but, at the very least, they do provide encouragement for further study of the *P. sidoides* extract's mode of action in anti-infective assays.

### 2.9. Release of Antimicrobial Peptides

Polymorphonuclear neutrophils (PMNs) play a key role in the front-line defence against pathogens. In addition to recruiting and activating other immune cells, PMNs have been demonstrated to release soluble peptides with broad-spectrum antimicrobial activity such as the bactericidal/permeability-increasing protein (BPI) and the defensins, human neutrophil peptides (HNP) 1–3. Recently EPs^®^ 7630 was shown to enhance the release of these antimicrobial peptides in a concentration-dependent manner [81]. At 30 μg/mL EPs^®^ 7630 increased the levels of HNP 1–3 and BPI by 150% and 127%, respectively. It should be noted that the extract was a much better inducer for the release of the defensins than LPS (82%, 10 ng/mL), whereas LPS proved to be more effective for BPI (356%). Interestingly, the combination of both stimuli enhanced the release of the studied antimicrobial peptides disproportionately (531% and 294% for BPI and HNP 1–3, respectively). This finding provided further evidence that the immune system reacts faster and most likely more effective against pathogens under the treatment of EPs^®^ 7630 than in the absence of this herbal medicine.

## 3. Conclusions

A traditional South African herbal medicine that has been used for centuries in the empirical treatment of respiratory tract diseases has found its way into European laboratories. Recent chemical, pharmacological and clinical studies of a special extract, referred to as EPs^®^ 7630 and developed from this traditional medicine, proved its efficacy in these conditions. Studying herbs such as *P. sidoides* for effects on the non-specific immune system may lead to the identification of novel chemical structures on which newer therapeutic agents can be derived and provide additional insight into the mode of action at the molecular level. This example provides a compelling argument for continuing the exploration of Nature and traditional medical systems as a source of therapeutically useful herbal medicines which satisfy current criteria of quality, safety and efficacy for an evidence-based therapy.
